# Can digital village construction promote sustainable agricultural development in China?

**DOI:** 10.1371/journal.pone.0329206

**Published:** 2025-08-06

**Authors:** Yuanchun Yu, Routing Zhang

**Affiliations:** School of Management, Sichuan University of Science and Engineering, Zigong, China; University of Illinois at Urbana-Champaign, UNITED STATES OF AMERICA

## Abstract

Agriculture is a major contributor to global greenhouse gas emissions. Consequently, studying the sustainable development of agricultural activities is crucial for achieving the United Nations’ Sustainable Development Goals. Utilizing panel data from 30 Chinese provinces from 2012 to 2022, this study measured the environmental, economic, and social dimensions of sustainable agricultural development (SAD) at the provincial level in China. Employing two-way fixed-effects and mediated effects models, the study empirically examined the driving effect of digital village construction (DVC) on SAD, along with the underlying mechanisms. The results reveal that the overall level of SAD in China has shown a gradual upward trend, although a pattern of higher SAD levels in the eastern regions compared to the west remains evident. DVC was found to exert a significant positive effect on SAD. Crucially, the market-based allocation of factors and agricultural product circulation were identified as significant mediating variables in this relationship. Heterogeneity analysis showed that the promoting effect of DVC is more significant in major grain-producing areas and in regions exhibiting higher SAD levels. Based on these findings, the study proposes targeted policy recommendations to provide practical strategies for different regions to advance DVC, narrow regional disparities, and enhance SAD levels.

## Introduction

The global agri-food system is suffering from unprecedented challenges related to food security and farmer livelihoods that profoundly affect environmental sustainability. Approximately 20–40% of the world’s land is degraded, thereby directly affecting the quality of life of nearly half of the world’s population [1]. Agricultural activities, as major sources of global greenhouse gas emissions and biodiversity loss [2], are placing increasingly significant pressure on the environment. Therefore, the goals outlined in the United Nations 2030 Agenda for Sustainable Development to eradicate hunger, achieve food security, improve nutrition, and promote sustainable agriculture are particularly crucial.

As the world’s largest grain producer, China plays a vital role in global food security [3], with output nearing 700 million tons in 2023 (≈25% of global production). However, its traditional agricultural model has caused severe environmental issues, notably agricultural surface source pollution due to the excessive use of pesticides and irrational utilization of agricultural waste [4], threatening social-economic and environmental sustainability [5]. Therefore, an eco-efficient agricultural development model is urgently required.

Research on SAD factors spans micro-level studies (e.g., nanotechnologies [6–8], microbial communities [9–11]) and macro-level analyses (e.g., human capital [12], industrial agglomeration [13], policy incentive [14], green innovation [15]). While digital technologies are now key drivers of agricultural transformation [16], their role in SAD remains under-explored. Therefore, this study addresses this gap by examining China’s digital village strategy, launched in 2018 to modernize agriculture and rural areas through digitalization, aiming to bridge the urban-rural divide, boost productivity, and promote SAD [17].

Despite existing research on DVC’s impacts, such as enhancing farmer education [18], income [19–21], and agroecological efficiency [22–25], three limitations persist: First, there remains a lack of holistic assessment of DVC’s overall effect on SAD. Second, the mechanisms linking DVC to SAD outcomes remain inadequately clarified. Third, existing heterogeneity analyses predominantly rely on economic or geographic factors [26,27], overlooking critical variations in agricultural characteristics.

In view of these limitations, this study contributes to the literature in three ways. First, it constructs a SAD evaluation index system from the environmental, economic, and social dimensions, objectively assessing SAD in 30 provinces in China with spatiotemporal characteristics to aid policy-making. Second, it integrates DVC and SAD into a unified analytical framework, revealing their nonlinear relationship, elucidating the dual-channel mechanism of market-based allocation of factors and agricultural product circulation, and enriching the discussion on digital-based SAD transformation mechanisms. Finally, it explores the heterogeneity of agricultural characteristic factors to enhance the scientificity and effectiveness of relevant policies.

## Theoretical analysis and research hypothesis

In DVC, technologies such as the Internet of Things, big data, and artificial intelligence provide intelligent precision management tools for agricultural production. These technologies realize real-time monitoring and precise management of crop growth and pest control [28], concurrently enhancing agricultural green total factor productivity and product quality [29]. Moreover, by enabling real-time monitoring of external ecological dynamics, DVC empowers farmers to implement timely environment protection measures and mitigate agricultural pollution, thereby advancing ecological sustainability [23]. Therefore, we propose hypothesis 1:

H1: DVC positively affects SAD.

In DVC, data as a new production factor, are formally included in the market-oriented allocation category. Data interconnection and sharing mechanisms optimize resource allocation in agricultural systems and enhance production efficiency. Concurrently, DVC elevates the marketization level of traditional production factors, such as land, labor, and capital [30]. Land transfer facilitation, labor market development, and financial innovation empower agricultural producers to flexibly access resources and promote the scale and intensification of development. These structural improvements inspire producers to pursue technological and management innovations, thereby reducing resource waste, enhancing resource efficiency, and contributing to SAD. Therefore, we propose hypothesis 2:

H2: DVC positively affects SAD by promoting the market-based allocation of factors.

The advancement of DVC has catalyzed significant improvements in rural information infrastructure, establishing a critical foundation for digital transformation within agricultural product circulation. Rural information service platforms provide farmers with timely and accurate market information and transaction channels, reducing information asymmetry [31]. The e-commerce platforms integrate supply chain resources to enhance product value-addition and market competitiveness [32], while cold-chain logistics reduces losses in the circulation process and guarantees quality and safety [33]. Improving the agricultural circulation system promotes agriculture and information technology integration, improves production efficiency and quality, and promotes rural economy diversification and SAD [34]. Therefore, we propose hypothesis 3.

H3: DVC positively affects SAD by promoting agricultural product circulation.

**Fig 1:**
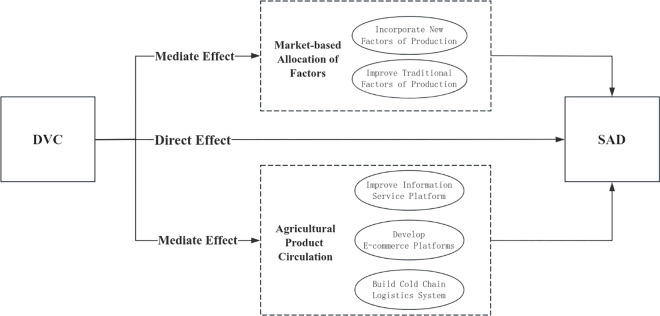
Theoretical model of the impact mechanism of DVC on SAD.

Fig 1 illustrates the theoretical model diagram.

## Materials and methods

### Methods

#### Entropy-weighted TOPSIS method.

The Entropy-weighted method determines the weight of the index according to its influence. The TOPSIS method measures the relative merits of each evaluation object by calculating its distance from the ideal solution and negative ideal solution. Combining the entropy-weighted method with the TOPSIS method can overcome the subjective nature of TOPSIS evaluations and objectively evaluate the development level of SAD in China. The calculation steps are as follows.

(1) Normalize the data by the extreme value method

In the multi-indicator evaluation system, different scales are applied to each evaluation indicator to eliminate the influence of scale. In this process, the data are first standardized as follows:


$$\text{\{Positive\textbackslash{} Indicators}:Y{\}}_{ij}=\frac{x_{ij}-x_{imin}}{x_{imax}-x_{imin}};\text{Negative\textbackslash{} Indicators}\;:Y_{ij}=\frac{x_{imax}-x_{ij}}{x_{imax}-x_{imin}}$$ (1)

Where $Y_{ij}$ is the standardized data for the indicator; $x_{ij}$ is the raw data for the j_th_ indicator in the i_th_ region; and $x_{imax}$ and $x_{imin}$ are the maximum and minimum values for the j_th_ indicator, respectively.

(2) Apply the entropy-weighted method to determine the weight of indicators

Calculate the share of the ith sample value under the jth indicator $P_{ij}$:


$$P_{ij}=\frac{X_{ij}}{\sum_{i=1}^{n}X_{ij}}$$ (2)

Calculate the information entropy of each indicator $E_{j}$:


$$E_{j}=-\frac{1}{lnn}*\sum {\mathrm{\backslash nolimits}}_{i=1}^{n}(P_{ij}*ln\left(P_{ij})\right)$$ (3)

Calculate the information utility value $D_{j}$:


$$D_{j}=1-E_{j}$$ (4)

Calculate the weighting factor *W*:


$$w_{j}=\frac{D_{j}}{\sum_{j=1}^{m}D_{j}}$$ (5)

Construct the weighting matrix *V:*


$$V={\left(v_{ij}\right)}_{n\times m}={\left(W_{j}\times H\right)}_{n\times m}$$ (6)

(3) Apply the TOPSIS method to calculate the level of SAD

Determine the positive and negative ideal solutions for each indicator:


$$\begin{array}{c} \text{Positive\textbackslash{} ideal\textbackslash{} solution}V^{+}=max\left[v_{1j},v_{2j},\cdots ,v_{ij}\right]\;; \end{array}$$

$$\text{Negative\textbackslash{} ideal\textbackslash{} solution}V^{-}=min\left[v_{1j},v_{2j},\cdots ,v_{ij}\right]$$

Use Euclidean distance to calculate the distance from each year to both solutions:


$$\begin{array}{c} D_{i}^{+}=\sqrt{\sum {\mathrm{\backslash nolimits}}_{j=1}^{m}\left(v_{ij}-V_{j}^{+}\right)};D_{i}^{-}=\sqrt{\sum {\mathrm{\backslash nolimits}}_{j=1}^{m}\left(v_{ij}-V_{j}^{-}\right)} \end{array}$$ (8)

$${CalculatetheproximityofeachyeartothepositiveidealsolutionC}_{i}:C_{i}=\frac{D_{i}^{-}}{D_{i}^{+}+D_{i}^{-}}$$ (9)

Where $C_{i}$is the closeness of the ith year, and the value range is [0,1]. A larger value of $C_{i}$ corresponds to a higher level of SAD, and *vice versa*.

#### Benchmark regression model.

Before proceeding with model selection, this study conducted the Hausman test to compare the applicability of the fixed effects model and random effects model during panel data analysis. The test results showed that the estimated coefficients of the random effects and fixed effects models did not significantly differ (*p* > 0.05), that is, both models can be used. However, based on the characteristics of the panel data, regressing the direct effects with a fixed-effects model can solve the bias due to the omission of variables. Further, controlling for individual fixed effects and time-fixed effects can eliminate unobservable heterogeneity between regions and trend factors as a function of time. Consequently, this study adopted a two-way fixed-effects model to test the direct effects of DVC on SAD. The following benchmark regression model was constructed:


$${SAD}_{it}=\alpha_{0}+\alpha_{1}{DVC}_{it}+\alpha_{2}{Control}_{it}+\mu_{i}+\nu_{t}+\varepsilon_{it}$$ (10)

where *i* represents the region, and *t* represents the year. *Control* is an ensemble of control variables. $\mu_{i}$ represents the individual fixed effects,$\nu_{t}$ is the time-fixed effect, and $\varepsilon_{it}$ is the random perturbation term.

#### Mediating effect model.

The direct impact of DVC on SAD was tested using Eq (10). Based on the theoretical analysis in the previous section, the mechanism underlying the impact of DVC on SAD was tested, and the mediating effect model was constructed, as shown in Eq (11).


$${Mediation}_{it}=\beta_{0}+{\beta^{\prime }}_{1}{DVC}_{it}+\beta_{2}{Control}_{it}+\mu_{i}+\nu_{t}+\varepsilon_{it}$$ (11)

where *Mediation* denotes the mediating variable, that is the market-based allocation of factors and agricultural product circulation. The remaining variables are consistent with those in Eq (10).

### Variable selection

#### Explained variables.

The explanatory variable in this study was the level of SAD, measured using a system of evaluation indicators. Sustainable development is defined as development that “meets the needs of the present without compromising the ability of future generations to meet their own needs” [35]. In agriculture, sustainability is the ability of farmers to continue harvesting crops and animal products without damaging the environment or resource base while maintaining economic profitability and social stability [36]. SAD is usually evaluated by constructing an evaluation system based on environmental, economic, and social dimensions [37–40], although several studies have further refined this system into a ternary framework of agricultural production–rural environment–farmer well-being and integrated resource input return dimensions [27,41]. SAD, grounded in sustainability, emphasizes that agricultural development must reasonably utilize natural resources, protect and improve the ecological environment, and continuously improve production and farmer income levels. Moreover, it must reduce rural poverty to foster sustained, stable, and comprehensive development of agriculture and rural economies. Therefore, this study measured SAD using environmental, economic, and social dimensions (see S1 Appendix for detailed indices).

The adverse characteristics of certain environmental indicators lie in their increasing values imposing pressure on ecosystems. Elevated application rates of fertilizers, pesticides, and plastic film per unit area directly impair soil health through pathways such as pollution, acidification, microplastic accumulation, and biodiversity loss, while also posing risks to ecosystem water quality. An increasing proportion of disaster-affected area signifies reduced land security, heightening susceptibility to erosion, degradation, and diminished resilience. Rising per capita electricity consumption is primarily detrimental when the energy mix is fossil fuel-dominated, leading to increased greenhouse gas emissions and resource depletion. Excessively high agricultural water use proportion depletes excessive groundwater and river resources, consequently reducing water flows available for ecosystems and other uses. These cumulative pressures erode the environmental foundation for sustainable development.

#### Explanatory variables.

The core explanatory variable is DVC. According to the DVC Guide 2.0, DVC encompasses eight dimensions: rural digital infrastructure, agriculture-related data resources, smart agriculture, rural digital enrichment industry, rural digital culture, rural digital governance, rural digital beneficiary services, and smart and beautiful villages. Based on the measurability of each dimension’s characteristics, we selected the rural digital enrichment industry, rural digital infrastructure, rural digital governance, and rural digital beneficiary service as primary indicators. Integrating the specific content of each dimension’s construction framework, we established corresponding secondary indicators and constructed the evaluation index system (Table 1).

**Table 1:** Indicator system for measuring DVC.

Primary indicators	Secondary indicators	Measurement indicators	Property
Rural Digital Enrichment Industry	Digital finance	Degree of digital finance digitization	+
	Digital industry	Gross value of rural e-commerce	+
	Digital investment	Digital Investment Business	+
Rural Digital Infrastructure	Internet penetration	Internet access subscribers	+
	Rural computer penetration rate	Computers per 100 rural households	+
	Penetration rate of mobile communication devices in rural areas	Mobile telephones per 100 rural households	+
Rural Digital Governance	Government digital governance	Government Digital Focus	+
	Market digital governance	Number of companies in the digital industry	+
	Social digital governance	Digital Insurance Business	+
Rural Digital Citizen Service	Breadth of digital financial coverage	Breadth of digital financial coverage	+
	Breadth of physical financial coverage	Number of financial institution outlets	+
	Level of network payments	Level of rural mobile payments	+

#### Control variables.

With reference to previous studies [42,43], this study included the following control variables: the level of economic development (GdpPC), which is expressed as the logarithm of GDP per capita; the level of transportation infrastructure (TraInf), which is expressed as the logarithm of highway mileage; the level of the labor force (Labor), which is expressed as the logarithm of the number of employed persons; the structure of the industry (IndStr), which is expressed as the output value of the tertiary industry/secondary industry; and the level of human capital (HR), which is expressed as the ratio of the number of students enrolled in tertiary schools to the total population.

#### Mediating variables.

The market-based allocation of factors (Market), references to the Report on China’s Sub-Provincial Marketization Index, constructs the evaluation index system presented in Table 2 according to the three aspects of capital, labor, and science and technology. The agricultural product circulation (ProCir) constructs the evaluation index system presented in Table 3 from the three aspects of the scale, efficiency, and organization of agricultural product circulation.

**Table 2:** Indicator system for measuring market-based allocation of factors.

Primary indicators	Measurement indicators	Property
Marketization of Capital Factors	Proportion of non-state and collective investment in total fixed asset investment in society	+
Marketization of Labor Factors	Number of persons employed in private and self-employed enterprises as a proportion of total employment	+
Marketization of Science and Technology Factors	Total amount of contracts transacted in the technology market as a share of R&D expenditure	+

**Table 3:** Indicator system for measuring agricultural product circulation.

Primary indicators	Secondary indicators	Measurement indicators	Property
Circulation Scale	Gross value of agricultural distribution per capita	Sales of wholesale and retail enterprises of agricultural products above the quota/total population at the end of the year	+
	Capitalization of the agricultural distribution industry	Total fixed assets/average gross output value of wholesale and retail enterprises of agricultural products above the quota level	+
Circulation Efficiency	Agricultural distribution cost ratio	(selling+management+financial) Expenses/main business revenue of agricultural products from wholesale and retail enterprises above the limit	+
	Profitability	Profit from main business/revenue from the main business of agricultural products from wholesalers and retailers above the quota level	+
Organization of Circulation	Turnover of the comprehensive agricultural products trading market	Turnover of the comprehensive agricultural products trading market	+
	Quality of agricultural product distribution personnel	Percentage of technical staff in agricultural products wholesale and retail enterprises above the quota level	+

#### Tool variables.

Owing to the possibility of bidirectional causality or omitted variables between DVC and SAD, this study mitigated endogeneity bias by selecting instrumental variables that only affect SAD through DVC.

The selection of instrumental variables(IVs) must satisfy the relevance and exogeneity conditions. We used the number of fixed-line telephones (Phones) per province in 2002 as the base instrumental variable [44]. As an early foundational communications infrastructure, fixed-line telephones directly enable internet adoption pathways upon which DVC critically depends, establishing relevance. The number of fixed-line telephones in 2002 peaked before the popularization of mobile telephones in 2003, exhibiting decaying “historical inertia” in the subsequent development of the Internet. This temporal precedence ensures exogeneity: while the instrument indirectly affects contemporary DVC, it exerts a negligible direct effect on current SAD outcomes. Given the cross-sectional nature of the base IV, we construct a panel IV following Nunn and Qian’s approach [45]. Specifically, we generate a time-varying IV through the cross-multiplier of each province’s 2022 fixed-line telephones and national IT service revenue to avoid the reverse causality problem.

### Data sources

Data were obtained from the 2012–2022 China Statistical Yearbook. Based on the completeness and reasonableness of the data, we selected 30 provinces, excluding Tibet, Hong Kong, Macao, and Taiwan. The results of the descriptive statistics for each variable are provided in Table 4.

**Table 4:** Descriptive statistics of variables.

Variables	Mean	Std. Dev.	Min	Max	Obs
SAD	0.3055	0.0788	0.1800	0.5370	330
DVC	0.2939	0.1590	0.0840	0.7940	330
GdpPC	10.9078	0.4446	9.8494	12.1547	330
TraInf	11.7144	0.8524	9.4368	12.1547	330
labor	7.6005	0.7675	5.5452	8.8639	330
Indstr	1.3844	0.7505	0.6100	5.2800	330
HR	0.0213	0.0057	0.0085	0.0436	330

## Empirical analysis and results discussion

### Calculation results of SAD

**Fig 2:**
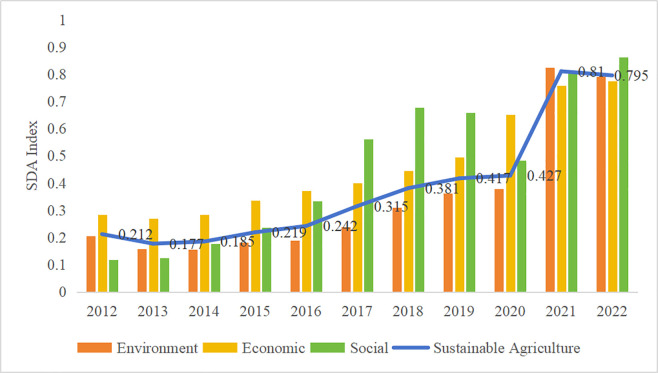
Evolution of China’s SAD index and its dimensional components (2012-2022).

The SAD indices for China and each province were obtained using the entropy-weighted TOPSIS method. Overall, the level of SAD in China showed a gradual upward trend, with especially significant growth from 2020 to 2021. However, prior to 2020, China’s SAD index was below 0.5 (Fig 2). The environmental effect of SAD corresponds with the overall development trend. The growth in 2021 was particularly significant, as the economic effect was increasing, while the development of the social effect fluctuated.

For a long time, China has been committed to promoting SAD. In 2020, China released a series of policies on green production in agriculture and the protection and utilization of agricultural resources. The measurements revealed that the series of policies have been well received. However, the small drop in 2022 shows that more support is needed to keep improving SAD

**Fig 3:**
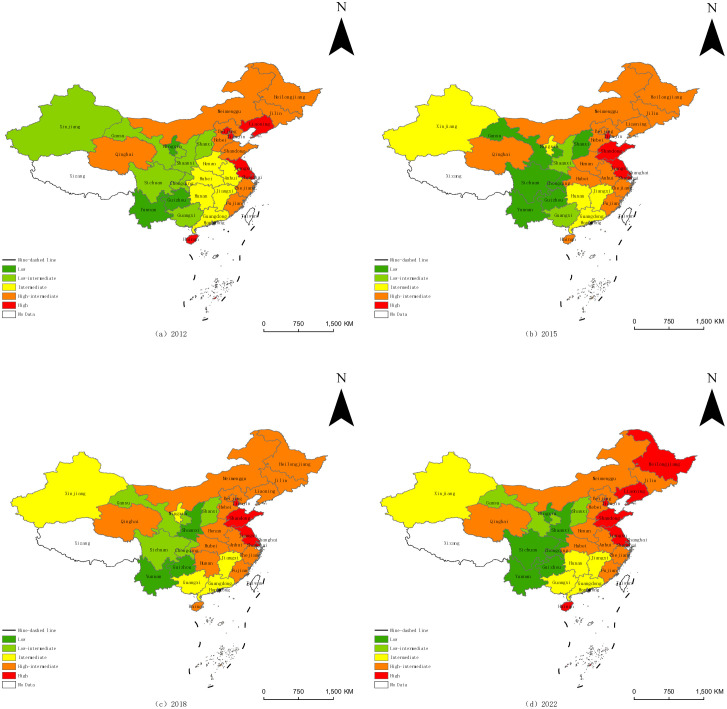
Evolution of spatial patterns of SAD, 2012, 2015, 2018, and 2022. (The base map outline was obtained by using ArcGIS based on the China National Platform for Common GeoSpatial Information Service (https://www.tianditu.gov.cn/), and the permission number is GS(2024)0650).

To better observe and compare the trends of SAD in China, this study used ArcGIS software, combined with the natural breakpoint method, to map the spatiotemporal dynamic characteristics of SAD. SAD in China exhibited an increasing trend, with significant increases in high-intermediate and high-level provinces (Fig 3). SAD exhibited an increasing trend from west to east, as indicated by the location of high levels of SAD in the eastern coastal region, and locations of low levels in the western region.

While Eastern China’s advanced modernization correlates with elevated pollution, emissions, and resource consumption, the region benefits from compensatory geographical advantages conducive to agriculture: predominantly flat terrain, substantial precipitation, and extensive irrigation infrastructure create robust environmental foundations. Moreover, economic prosperity enables sufficient financial support for agricultural production, while convenient transportation increases market demand and improves the sale conditions of agricultural products. Conversely, Western China’s inland position manifests fundamental constraints: limited precipitation and high mountains and hills with fewer plains. These factors result in poor natural conditions for agricultural development, which increase the difficulty of constructing agricultural infrastructure and promoting green agricultural technology.

### Results of the empirical analyses

#### Benchmark regression.

We employed a two-way fixed-effects model to control for both individual and time effects. The benchmark regression results, presented in Table 5, indicate that DVC significantly promotes SAD. As shown in Column (2), a one-unit increase in the DVC index corresponds to a 0.158-unit increase in the SAD index (p < 0.05). This finding provides empirical support for China’s integrated policies advancing DVC and SAD. During the process of DVC, the application and promotion of emerging technologies such as the Internet of Things, big data, and artificial intelligence have provided innovative solutions for SAD. These technologies optimize the agricultural production process through precision monitoring, intelligent decision-making, and automated production processes, thereby enhancing resource allocation efficiency and reducing environmental pollution.

**Table 5:** Benchmark regression results.

	(1) SAD	(2) SAD
DVC	0.1549***	0.1580***
	(0.0305)	(0.0292)
GdpPC		0.0066
		(0.0190)
TraInf		−0.0355**
		(0.0154)
labor		0.0119
		(0.0159)
IndStr		−0.0374***
		(0.0063)
HR		2.5904***
		(0.6948)
_cons	0.2607***	0.5032**
	(0.0090)	(0.2243)
N	330	330
R²	0.1134	0.2855
YearFE	Yes	Yes
ProvinceFE	Yes	Yes

Notes: Standard errors are in parentheses;^*^p < 0.1,^**^p < 0.05,^***^p < 0.01.

(Consistent with this note unless otherwise indicated).

Regarding control variables, the construction of transportation infrastructure has a negative impact on SAD. The increased development of transportation infrastructure entails large-scale occupation of arable land resources. Moreover, improved transportation conditions increase the number of automobiles. These dual mechanisms exacerbate air pollution and stain energy systems, thereby imposing a negative influence on SAD. Industrial restructuring similarly demonstrates adverse impacts. As regional economies transition toward secondary and tertiary sectors, agricultural investment experiences a relative decline due to capital reallocation, constraining SAD. Conversely, human capital accumulation emerges as a significant positive factor. The influx of skilled talent to rural areas facilitates knowledge spillovers and technological diffusion, directly enhancing agricultural productivity while advancing sustainable practices, constituting a primary channel for SAD improvement.

#### Robustness tests.

To verify the findings, this study employed a regression model comparison, excluded special samples, and utilized an instrumental variable method to test the robustness of the model.

(1) Model comparisons

This study compared the regression results of the random effects model with the regression results of the fixed effects model (Table 6(1)). The regression coefficients demonstrated that the driving effect of DVC on SAD was significant, as the coefficients did not differ significantly when using different models. Thus the results of this study are robust.

**Table 6:** Robustness test results.

	(1) Model Comparison		(2) Excluding Sample	(3) Instrumental Variable	
	Fe	Re		First stage	Second stage
VARIABLES	SAD	SAD	SAD	DVC	SAD
DVC	0.1580***	0.1519***	0.1547***		0.3703***
	(0.0292)	(0.0282)	(0.0537)		(0.1160)
Phone				0.0000***	
				(0.000)	
_cons	0.5032**	0.5359***	0.3269	−2.1451***	0.8685***
	(0.2243)	(0.2041)	(0.4589)	(0.4079)	(0.3150)
Controls	Yes	Yes	Yes	Yes	Yes
YearFE	Yes	Yes	Yes	Yes	Yes
ProvinceFE	Yes	No	Yes	Yes	Yes
Observations	330	330	286	330	330
Kleibergen-Paap LM					19.486***
Kleibergen-Paap Wald F					24.834 [16.38]^a^
Hansen J					/

Notes: ^a^Critical values at the 10% level of the Stock-Yogo test within [].

(2) Excluding special samples

Given the distinct structural characteristics of China’s municipalities, including economic structure, urbanization level, and policy inclination, which differ substantially from general provinces, the four municipalities (Beijing, Shanghai, Tianjin, and Chongqing) were excluded from the regression analysis to reduce heterogeneity interference. As presented in Table 6(2), the regression coefficients remained significant and exhibited no substantive divergence from baseline estimates, confirming the robustness of core findings to the exclusion of the special samples.

(3) Instrumental variables

The two-stage least squares regression results are presented in Table 6(3). The regression results demonstrate that the instrumental variables passed the unidentifiable (i.e., the Kleibergen–Paap LM statistic was significant) and weak instrumental variable tests (i.e., the Kleibergen–Paap Wald F statistic was greater than the critical value at the 10% level). A significant correlation exists between instrumental and explanatory variables in the first stage of regression. In second-stage regression, DVC exhibited a significant positive driving effect; thus the benchmark model had no serious endogeneity issues. This study did not employ the Hansen J test because only one instrumental variable was used to explain a single endogenous variable, resulting in an exactly identified model. The Hansen J test is applicable only in over-identified scenarios.

#### Mechanism analysis.

This section examines whether DVC affected SAD by promoting the market-based allocation of factors and agricultural product circulation. Table 7 presents the regression results of Eqs (10) and (11), demonstrating that DVC significantly promoted SAD and market-based allocation of factors, effectively reducing resource waste, improving resource utilization efficiency, and promoting SAD. Therefore, H2 is supported. Table 7 also reveals that DVC promoted the agricultural product circulation, thereby promoting the integration of agriculture and information technology, improving the efficiency and quality of agricultural production, and promoting SAD. Thus, H3 is supported.

**Table 7:** Mechanism analysis results.

	(1) SAD	(2) Market	(3) ProCir
DVC	0.1580***	0.3893***	0.1823**
	(0.0292)	(0.1236)	(0.0767)
_cons	0.5032**	1.5475	1.8267***
	(0.2243)	(0.9490)	(0.5893)
N	330	330	330
R²	0.2855	0.2344	0.4149
Controls	Yes	Yes	Yes
YearFE	Yes	Yes	Yes
ProvinceFE	Yes	Yes	Yes

### Heterogeneity analysis

(1) Heterogeneity of functional food production areas

As food is the foundation of human survival, an in-depth exploration of the differentiated impacts of DVC in promoting SAD in different functional food production zones is significant for the precise formulation of policies that maximize the advantages of each region and accelerate the pace of SAD. Thus, the research sample was subdivided into two clusters: major and non-major grain-producing areas. The different impacts of DVC in these two areas were systematically analyzed. The promotional effect of DVC was more significant in the major grain-producing areas (Table 8(1,2)), where it further improved the efficiency and quality of grain production as well as stabilized national grain supply and market prices.

**Table 8:** Heterogeneity analysis results.

	(1) Major areas	(2) Non-major areas	(3) High level	(4) Low level
	SAD	SAD	SAD	SAD
DVC	0.1210***	0.0735**	0.0863***	0.0686
	(0.0453)	(0.0354)	(0.0294)	(0.0866)
_cons	0.2656	1.0008***	0.6336	−1.2514***
	(0.3781)	(0.2720)	(0.4873)	(0.3924)
N	143	187	176	154
R^2^	0.4853	0.4656	0.5240	0.6050
Controls	Yes	Yes	Yes	Yes
YearFE	Yes	Yes	Yes	Yes
ProvinceFE	Yes	Yes	Yes	Yes

(2) Heterogeneity of SAD levels

According to the mean value of SAD, this study categorized regions into high or low levels of SAD to examine the heterogeneity of the impact of DVC. In regions with high levels of SAD, DVC significantly promoted SAD (Table 8(3,4)). Agricultural production already has a certain modernization and intelligence foundation in regions with a high level of SAD. These areas typically have an optimal agricultural infrastructure, advanced agricultural technology, and higher-quality farmers. This study provides a solid foundation for the promotion of DVC, making the application of digital technology in agricultural production smoother and more efficient, thereby promoting SAD.

## Discussion

This study confirms that DVC significantly promotes SAD, consistent with existing literature on digitalization’s positive agricultural role [23,26,29]. Our findings further reveal that DVC drives SAD through two specific channels: enhanced market-based allocation of factors and efficient agricultural product circulation. This mechanism addresses a gap in prior research, which overlooked these pathways in analyzing SAD influencing factors.

The SAD evaluation system developed in this study differs from earlier approaches that focused narrowly on isolated dimensions like pollution reduction or resource efficiency [27,41]. By integrating environmental, economic, and social dimensions, our framework provides a more comprehensive assessment of China’s SAD status, better reflecting systemic interconnections and offering improved policy reference.

Regarding heterogeneity, this study differs from prior regional analyses [46,47] by focusing on agricultural characteristics. We find stronger DVC effects in major grain-producing areas, contrasting with Wang et al. [27] and Li and Peng [48]. This outcome is attributed to scale-driven efficiency. Major grain-producing areas’ concentrated production enables effective digital technology integration, while non-major areas’ diversified operations lead to fragmented adoption and weaker impacts. Additionally, the differential effects observed across regions with high versus low SAD levels support context-specific policy design.

Despite the theoretical and empirical contributions, this study has limitations that need to be addressed in future research. On the one hand, given the short duration of China’s digital village pilot program, it is currently challenging to comprehensively and accurately assess the actual effects of the policy implementation. Future research should delve deeper into the policy effects of the digital village pilot program to provide more comprehensive results on its long-term impacts on rural development. On the other hand, due to data availability and completeness, this study uses data from 30 provinces in China. However, the focus of DVC should be on rural areas at the county level. Future research should enrich and collect relevant county-level data to more accurately analyze the actual impacts of the policy and offer more targeted recommendations.

## Conclusions and policy recommendations

Based on data from 30 provinces in China from 2012 to 2022, this study reviews the level of SAD and explores its spatial and temporal evolution characteristics. Dictionary, based on theoretical analyses, the total effect of DVC on SAD is empirically examined and the mechanism of action and heterogeneity are discussed. The main conclusions are as follows.

(1) In terms of the spatial and temporal development characteristics, the overall level of SAD in China presents a gradual upward trend, although the SAD index slightly declined in 2022; an imbalance is observed in the development of the eastern and western regions, with an overall trend of enhancement from west to east. To this end, in order to maintain the benefits of SAD, local governments need to consolidate the foundation for DVC, advance pilot programs for digital villages, and draw on the experience of advanced regions. Eastern regions should leverage their economic advantages and well-developed digital infrastructure to upgrade rural e-commerce platforms, expand sales channels for agricultural products, and increase their value-added; whereas western regions, given their relatively weak digital foundation and rich characteristic resources, should focus on strengthening digital infrastructure construction, integrating ethnic cultures, tourism, and other local resources into digital transformation initiatives. For example, developing digital tourism platforms and characteristic agricultural product e-commerce platform can enhance the visibility and competitiveness of characteristic industries, and attract tourists and investors.
(2) The empirical results show that DVC significantly promoted SAD, with every 1 unit increase in the DVC index significantly increasing the SAD index by 0.1580 units. The mechanism analysis shows that promoting market-based allocation of factors and agricultural product circulation is an important method by which DVC promotes SAD. Thus, localities should actively promote the market-based allocation of factors, improve the rural property rights system, strengthen the protection of the legitimate rights and interests of farmers, and stimulate farmers to participate in market transactions. In addition, localities should enhance the level of agricultural product circulation. Specifically, they need to establish an information platform dedicated to the circulation of agricultural products. This platform should facilitate the interconnection of information across various links in the agricultural products supply chain, including production, processing, and circulation.
(3) Heterogeneity analysis shows that the driving effect of DVC on SAD is more significant in major grain-producing areas and areas with high SAD level. Therefore, it is recommended to adopt differentiated strategies. For major grain-producing areas and regions with high SAD level, efforts should focus on deepening digital penetration across the entire agricultural value chain. This includes scaling up digital infrastructure, advancing precision agriculture, and deploying smart agricultural machinery, thereby maximizing the multiplier effect of digital technologies in sectors where these regions already excel.For non-major grain-producing areas and regions with low SAD level, priority should be given to systematically narrowing the digital gap. A phased mechanism integrating digital infrastructure construction, farmer digital skill training, and targeted industrial support is critical here. Government special funds can guide social capital to develop lightweight, locally tailored digital service platforms, while step-by-step skill training ensures farmers can effectively participate in digital transformation. These measures will lay the groundwork for DVC to drive SAD in these regions over time.

## Supporting information

S1 Appendix Indicator system for measuring SAD. (DOCX)
