# Supplementary material for: Can digital village construction promote sustainable agricultural development in China?
Source: PLoS One. 2025 Aug 6;20(8):e0329206. doi: 10.1371/journal.pone.0329206 (PMC12327646; doi:10.1371/journal.pone.0329206)
Supplement: S1 Appendix — (DOCX) [file pone.0329206.s001.docx]

**Table 9. Indicator system for measuring SAD**

| Primary indicators | Secondary indicators | Measurement indicators | Property |
| --- | --- | --- | --- |
| Environment | Soil quality | Fertilizer application per unit area | - |
|  |  | Pesticide application per unit area | - |
|  |  | Plastic film use per unit area | - |
|  | Land use | Percentage of cultivated land area | + |
|  |  | Percentage of forested land area | + |
|  |  | Number of State-level nature reserves | + |
|  | Land security | Percentage of area under soil erosion control | + |
|  |  | Percentage of waterlogged area | + |
|  |  | Percentage of affected area | - |
|  | Energy | Total power of agricultural machinery per unit area | + |
|  |  | Per capita electricity consumption | - |
|  |  | Renewable energy use | + |
|  | Water source | Average reservoir capacity | + |
|  |  | Percentage of irrigated area | + |
|  |  | Percentage of water used in agriculture | - |
| Economic | Production | Agricultural labor productivity | + |
|  |  | per capita yield of grain | + |
|  |  | Food production per capita | + |
|  |  | Meat production per capita | + |
|  |  | Per capita fish production | + |
|  | Output | Gross output value of agriculture, forestry, animal husbandry, and fishery as a percentage of GDP | + |
|  |  | Share of the total output value of agriculture, forestry, animal husbandry, and fishery services in total output value of agriculture, forestry, animal husbandry, and fishery services | + |
|  |  | Per capita value of agricultural, forestry, and fishery services | + |
|  | Support | Percentage of expenditure on agriculture, forestry, animal husbandry, and fishery affairs | + |
|  |  | Share of investment in fixed assets in agriculture, forestry, livestock and fisheries | + |
| Social | Social and livelihood development | Years of schooling per capita | + |
|  |  | Percentage of population with low income | - |
|  |  | Per capita disposable income of farmers | + |
|  |  | Engel's coefficient for rural areas | - |
|  | Basic livelihood security | urbanization rate | + |
|  |  | Water supply penetration | + |
|  |  | Gas penetration rate | + |
|  |  | Number of health rooms per 10,000 population | + |
